# Post operative temporary epicardial pacing: When, how and why?

**DOI:** 10.4103/0974-2069.43877

**Published:** 2008

**Authors:** Anjan S Batra, Seshadri Balaji

**Affiliations:** Department of Pediatric Cardiology, University of California Irvine, Orange, USA; 1 Oregon Health and Science University, Portland, USA

**Keywords:** Epicardial pacing, post operative, temporary

## Abstract

Temporary epicardial pacing is commonly used for the diagnosis and treatment of arrhythmias in the acute post operative period after surgery for congenital heart disease. Temporary epicardial pacemakers have become increasingly sophisticated over the years and have evolved from simple single chamber devices with few programmable parameters to complex dual chamber devices capable of adjustable parameters similar to permanent pacemakers. This review will describe the various indications for temporary pacing, technical considerations for both the choice of pacing wires and pacemaker modes, complications with temporary pacing and our current practice with temporary pacing.

Postoperative arrhythmias are a major cause of mortality and morbidity after cardiac surgery for congenital heart disease (CHD).[1] Patients with CHD are especially vulnerable to rhythm disturbances in the early postoperative period. The higher incidence of postoperative arrhythmias in this patient population has been attributed to multiple risk factors including a lower body weight,[2,3] longer cardiopulmonary bypass duration, and a higher surgical complexity. Temporary epicardial pacing wires are routinely placed after surgery for CHD and temporary pacing has become a useful and, at times, an essential modality for maintaining hemodynamic stability.

## INDICATIONS

### Diagnostic

Atrial pacemaker wires can be used to create an atrial electrogram (AEG) with augmented P waves. The recording is done by connecting the atrial pacemaker wires to the left and right arm leads. This creates a bipolar AEG in lead I and a unipolar AEG in the remainder of the limb leads. An alternative option is to attach the pacing lead to one of the precordial chest leads.

**Figure 1:**
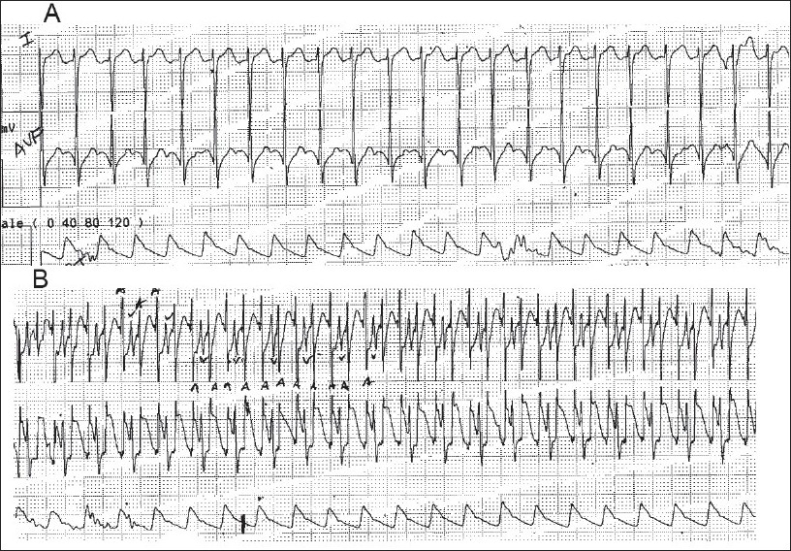
(A) Surface electrocardiogram of a patient in atrial flutter with 2:1 AV conduction. The P waves are not clearly seen, (B) Atrial electrocardiogram of the same patient shows the P waves with 2:1 AV conduction more clearly

**Figure 2:**
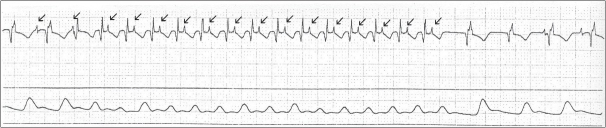
An atrial electrocardiogram showing onset and termination of a junctional tachycardia. The P waves are retrograde and marked with arrows

Diagnostic AEGs are useful when P waves are not clearly visible on a surface electrocardiogram (ECG) [Figure 1]. They also help to differentiate junctional tachycardia from supraventricular tachycardia and sinus tachycardia. In junctional tachycardia, the P waves are either superimposed on the R waves or dissociated from the R waves [Figure 2]. In supraventricular tachycardia the PR interval is longer than the RP interval, and in sinus tachycardia the PR interval is smaller than the RP interval. AEGs may also be useful in differentiating various degrees of heart block and sinus node dysfunction. In heart block, the atrial rate is generally faster than the ventricular rate as opposed to sinus node dysfunction where the reverse holds true.

### Therapeutic

**Figure 3:**
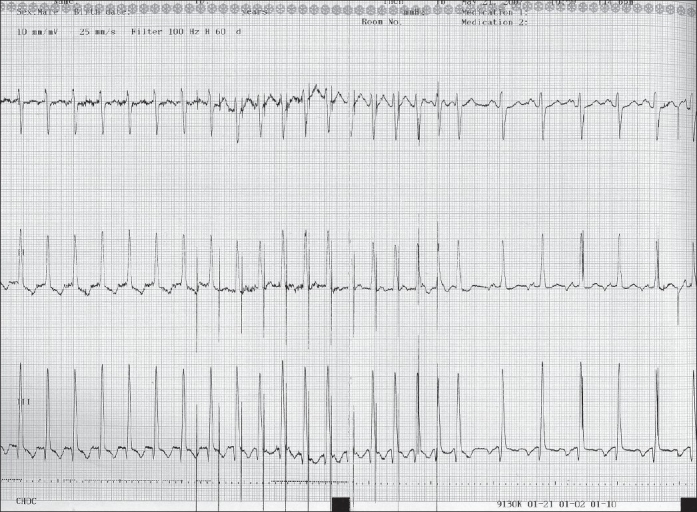
A 3 lead electrocardiogram: The first part demonstrates a slow atrial tachycardia at a rate of 140 beats per minute with 1:1 AV conduction, the second part rapid atrial pacing using a temporary pacemaker and the third part sinus rhythm after termination of the tachycardia

Sinus node dysfunction, junctional ectopic tachycardia, supraventricular tachycardia (AV nodal reentry, atrio-ventricular reentry, flutter and sinus node rentry tachycardia), and atrioventricular block are common postoperative arrhythmias[2,3] Temporary overdrive pacing can be an effective means of terminating reentry tachycardias such as atrial flutter [Figure 3] and paroxysmal supraventricular tachycardia.[4] Pacing for short durations generally takes over the circuit and subsequently terminates the tachycardia. Typically the pacing rate is set at 10-20 beats faster than the tachycardia rate. Progressively faster rates can be tried with multiple attempts.

In patients with junctional ectopic tachycardia, temporary dual chamber pacemakers can be used to establish AV synchrony by pacing the atrium at rates faster than the junctional rate.

Sinus node dysfunction and high grade heart block are not so uncommon complications of surgery for CHD. Over half these patients with conduction abnormalities will have normalization of conduction within 10 days from cardiac surgery. Temporary epicardial pacing can be used to bridge this interval between post operative heart block and either recovery of spontaneous conduction or placement of a permanent pacemaker. In extremely low-birth-weight infants with complete heart block, patient size may preclude implantation of a permanent pacemaker. Extrathoracic temporary epicardial pacing may provide support to these patients for up to three months at which time permanent, epicardial-pacing leads can be implanted.[5]

Disturbance of normal AV synchrony and dyssynchronous ventricular contraction may be deleterious in patients with CHD and compromised hemodynamics. Janousek *et al*, evaluated the effect of optimizing temporary dual chamber pacing in patients after surgery for congenital heart disease and demonstrated several techniques of individually optimized temporary dual chamber pacing to improve hemodynamics (as measured by a higher systolic arterial blood pressure and lower atrial pressure) by optimizing AV synchrony and/or synchronous ventricular contraction.[6]

### Programming

The pacing mode is classified using the Heart Rhythm Society and British Pacing and Electrophysiology Group generic code [Table 1].[7] The first three positions specify the type of communication taking place (sensing vs. pacing) and with which cardiac chamber (atrium vs. ventricle). Position IV specifies the presence or absence of rate modulation and is not programmable in temporary pacemakers. Position V specifies the location of multisite pacing (if applicable) i.e., biatrial or biventricular pacing.

**Table 1:** Heart Rhythm Society and British Pacing and Electrophysiology Group generic code

I	II	III	IV	V
Chamber paced	Chamber sensed	Response to sensing	Rate modulation	Multi site pacing
O = none	O = none	O = none	O = none	O = none
A = atrium	A = atrium	T = triggered	R = rate modulation	A = atrium
V = ventricle	V = ventricle	I = inhibited		V = ventricle
D = dual	D = dual	D = dual		D = dual
(A + V)	(A + V)	(T + I)		(A + V)

The single-chamber external pulse generators are easy to use and can be programmed in the VVI, VOO, AAI, or AOO mode. They also have an increased sensitivity range (0.5 to 20 mV) to provide increased ability to sense P- and R-waves over other devices. Modern day dual chamber temporary pacemakers allow adjustment of parameters such as mode of pacing, lower and upper rate limits, AV interval, and post ventricular atrial refractory period. The most important of these are rate, atrial output and ventricular output (in most commercially available temporary pacemakers, the duration of the electrical impulse, namely the pulse width is fixed and cannot be programmed by the physician). Making appropriate adjustments of these three parameters will provide effective pacing in most clinical situations. Changing the pacing rate automatically adjusts other dual-chamber temporary pacing parameters. Pediatric patients may need a pacing rate up to 200 ppm. A patient's high stimulation threshold may require a ventricular output of 25 mA. For managing atrial tachyarrhythmias, a rapid atrial pacing rate up to 800 ppm is possible but rates beyond 400/min are rarely used.

The optimal sensing and pacing parameters need to be adjusted based on the thresholds. The capture threshold is the minimum pacemaker output (current intensity measured as voltage or amperage) required to stimulate an action potential in the myocardium. Only the current amplitude (volts/amperes) is altered in measuring threshold since temporary pacemakers come with a fixed current duration (also termed pulse width). This should be checked on a daily basis especially in patients who are dependent on the temporary pacing. Typical default settings are to set the voltage outputs at twice threshold in both atrium and ventricle to allow for a margin of safety. This however may not be possible if the capture threshold is > 10 mA. The sensing threshold is the minimum current the pacemaker is able to sense. It is generally recommended to set the sensitivity at half the minimum current sensed. If sensing is set too high (insensitive) this will make the pacemaker fail to sense intrinsic events, thereby converting it from a demand to a fixed-rate pacemaker. Setting the sensitivity too low (over-sensitive) will make the pacemaker potentially sense muscle noise, and other ambient events common to the intensive care situation (such as ventilators, intravenous pumps etc) as intrinsic events leading to inappropriate loss of pacing. The upper and lower rate limits and AV intervals vary with age and should be set to maximize cardiac output within the range of age appropriate physiological parameters. In general, the AV delay is set between 100-140 ms in most children.

Newer applications of temporary pacemakers include their use for the simultaneous pacing of both ventricles. Both ventricles can be simultaneously paced by placing a right and a left ventricular lead at implant and connecting both wires to the same output terminal of the pulse generator. There is evidence to show that biventricular pacing can augment cardiac performance in patients after repair of CHD and that this form of pacing may be better than conventional RV pacing in patients with normal interventricular conduction.[8–10] On a practical note, it is important to check the threshold for each ventricle and set the ventricular output above the higher of the two thresholds so that both ventricles can be successfully paced. It is also important to remember that the negative electrode (cathode) is the active one and that the positive pole (anode) is the inactive/indifferent electrode.

## PACING WIRES

### Polarity

Epicardial wires come in two forms: unipolar and bipolar. A unipolar system [Figure 4A] consists of the negative cathode attached to the epicardium and the positive anode attached to the subcutaneous tissue. The pediatric unipolar lead is designed for thinner pediatric and atrial tissue by being smaller and having a curved chest needle for better leverage through small chest cavities. The larger current in a unipolar system creates a larger pacing spike on the surface ECG. It also has a cost advantage over the bipolar pacing lead. A bipolar system [Figure 4B] consists of a single wire with both the anode and the cathode on this wire attached to the epicardial surface. The cathode is usually the more distal electrode and the anode is 8 mm proximal to the cathode (the Medtronic bipolar coaxial 6495: Medtronic, Minneapolis, MN). Because of the shorter distance between the two poles in the bipolar lead, the capture threshold is generally lower and the sensing better when compared to the unipolar lead.

**Figure 4A:**
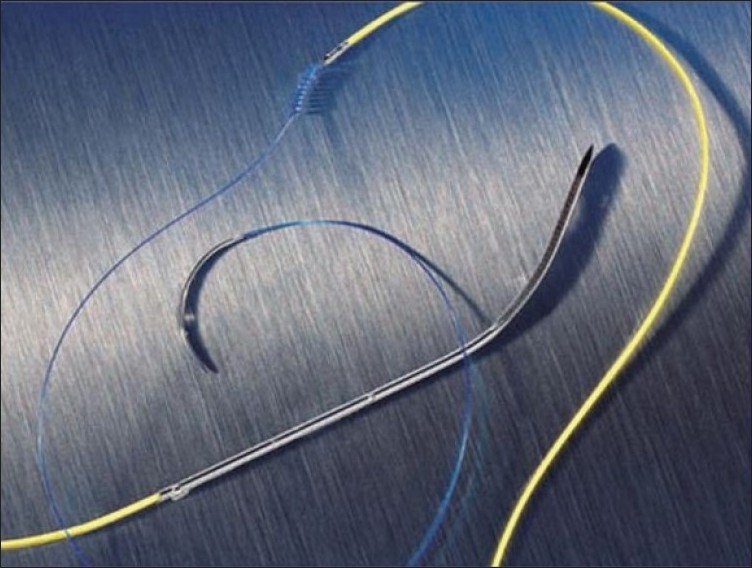
Shows model 6491 unipolar pediatric temporary pacing lead

**Figure 4B:**
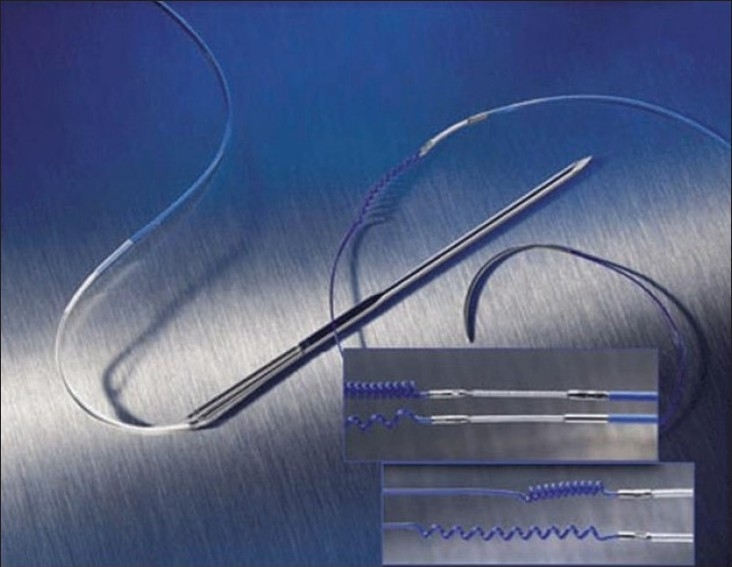
Shows model 6495 coaxial bipolar temporary myocardial pacing lead. Two discreet electrodes are optimally spaced at 8mm for consistent pacing and sensing

### Insertion of leads

Temporary epicardial pacing leads are typically placed in the operating room after the cardiac procedure is completed and before chest closure. Epicardial pacing wires were historically placed only on the right ventricle. This allowed for management of severe bradyarrhythmias with a more stable ventricular rate but at the expense of AV synchrony. As patients with CHD are often dependent on the atrial contraction for better preload and cardiac output, most institutions currently place both atrial and ventricular pacing wires. The atrial wires are placed on the right atrium.

By informal convention, wires placed on the right atrium are brought out through the skin on the right of the sternum, and those on the right ventricle are brought out on the left of the sternum. The wire connected to the negative terminal is marked by color coding.

An implanted pacing lead forms a direct current path to the myocardium. During pacing lead insertion and testing, only battery-powered equipment specially designed for this purpose should be used to protect against fibrillation that may be caused by alternating currents. Line-powered equipment used in the vicinity of the patient must be properly grounded. Pacing lead connector pins must be insulated from any leakage currents that may arise from line-powered equipment. Care should be taken to avoid the possibility of unintentional contact between the pacing lead(s), including extension cable, and any equipment used as well as any conductive surface contact.

### Duration

Although both atrial and ventricular temporary epicardial leads are reliable for short-term use, their function deteriorates on a daily basis. These leads are intended for temporary atrial and ventricular pacing and sensing for contemplated implant duration of 7 days or less. Increase in stimulation threshold typically occurs after 4 days in both the atrial and ventricular wires.[11] The reason for increasing threshold is believed to be secondary to inflammation around the surface of the myocardium where the wire is attached. As bipolar electrodes require less energy for capture, they have a longer longevity when compared to a unipolar system.[12,13] Temporary epicardial pacing leads have been used for periods of up to 3 months when patient size or other factors may preclude implantation of a permanent pacing system.[5] Steroid eluting endovascular pacing wires are available for use with permanent pacemakers and significantly reduce the inflammation around the myocardium where the wire is attached. To date, steroid eluting temporary pacing wires have not been developed.

### Complications of temporary pacing

There is a small but definite risk of epicardial pacing. Complications of epicardial wires include infection, myocardial damage, ventricular arrhythmias, perforation, and tamponade.[14,15] While temporary pacing leads are removed by gentle traction, ECG should be monitored (observing for arrhythmias) and the patient should be observed for a few hours after lead removal because of the risk of tamponade. Leaving the lead implanted for longer than 7 days may result in difficulty or inability to extract the lead and/or bleeding.

The use of MRI is currently not recommended in patients dependent on temporary epicardial pacing because of the potential risk of precipitating an arrhythmia[16] or causing injury from excessive heat at the electrode tip.[17] An MRI may however be safely performed in a patient with retained epicardial wires that have been cut off at the skin because of the absence of an antennae capable of concentrating the energy from the MRI.[18]

### Alternatives to temporary epicardial pacing

In certain cases where epicardial pacing wires are not available or fail to function, alternative means of pacing need to be considered. These include pacing through transcutaneous patches, a transvenous lead or a transesophageal lead. Transcutaneous patches are readily available in the intensive care units and can be used to initiate asynchronous ventricular pacing in a relatively short period of time. Transcutaneous pacing has many limitations including making the patient extremely uncomfortable, requiring high energies for capture, and is generally not recommended for periods over 24 hours. A transvenous pacing lead can be inserted using fluoroscopic guidance and typically used to stimulate ventricular depolarization. Although more reliable than the transcutaneous patches, there is a risk of lead dislodgement and ventricular perforation Transesophageal pacing is used for brief bursts to overdrive the atrium in order to terminate atrial tachycardias. Ventricular capture is not possible and hence this form of pacing is not useful for heart block. There is also the risk of esophageal damage when used for longer periods of pacing at high output.[19] There have been some animal studies where transesophageal pacing has been shown to be safe and effective for periods of up to 24 hours.[20] Permanent pacemaker implantation is indicated if the need for a temporary pacemaker persists longer than 10-14 days postoperatively.[21]

### Current practice

Our current practice involves placement of atrial and ventricular unipolar pediatric pacing wires in all patients undergoing surgery for CHD who have undergone cardiopulmonary bypass. The risk of arrhythmias is considered low in patients that do not undergo cardiopulmonary bypass and hence temporary pacing wires are routinely not placed in these patients. Temporary pacing is undertaken to treat dysrhythmias and to improve hemodynamics in select patients with heart failure. Patients with complete heart block are paced using the temporary wires for 10-14 days for their underlying rhythm to return before placing a permanent pacemaker. The temporary epicardial leads are removed just before the patient is discharged from the intensive care unit.

Fishberger *et al*,[22] have recently shown that not all patients undergoing surgery need temporary pacing wires and that pre and intraoperative parameters can help predict the need for pacing wires. Their approach, while attractive, needs to be validated by other centers before it can be uniformly adopted.
